# Bacteriology

**Published:** 1887-10-01

**Authors:** 


### THE DOCTOR'S PULPIT.

### BACTERIOLOGY.

Everyone who knows by experience those peculiar difficulties in medical practice which want of complete knowledge puts in the way of the practitioner will be in the utmost sympathy with the work of original research. If a machine, of whose construction its owner is to some extent ignorant, gets out of order, he does not know how to set about mending it. If a chemical process be not thoroughly understood it may entirely fail of its issue on account of some more or less serious mistake, without the operator having the power to prevent or correct the fault. If results are to be exact and uniform, processes must be understood. Whilst there is darkness and doubt in the method there must be uncertainty in the finished work.

A great deal of medical practice fails of a successful termination because medical men are so much in the dark. They often do not know in any full and perfect measure the cause and nature of the diseases they have to treat, nor do they understand with clearness and accuracy the actual effect of many of the medicaments they use. But that is, in most cases, their misfortune, not their fault. The best of them are anxious—even painfully anxious—to master thoroughly the secrets of disease ; and it is these who are constantly striving by every method which has previously been tried, and every additional means their own ingenuity can devise, to find out exactly and entirely the real origin and nature of those physical ills which we call diseases, and which so strangely affect mankind. That unconquerable and laudable curiosity is the parent of modern research, and modern research offers to us a "germ" theory of disease. It is not necessary, nor would it be interesting, to give an account of leading theories which have preceded this in the history of medical progress. They have been numerous, and sometimes perhaps even foolish. There is this to be said in favour of the permanency of recent theories as against those of more remote times, that the methods of modern days are such as tend to the discovery of facts on which theories can be based. They are not the same as many of those employed by men of former generations. They too often elaborated a theory in the study, and then tried to make facts square with it. The moderns carefully ascertain their facts first, and when they have obtained a sufficient number, they make a generalisation which shall express the law or laws underlying the facts. The modern method builds the house of whatever materials it can find, and when it is finished the builder gives it such a name as its characteristics manifestly demand. If it has the appearance and attributes of a palace he calls it a palace ; but if in look and character it is clearly a pigstye he does not hesitate to put a "pigstye" label upon it. The ancients, and not they only, too often named their "theoretical" house before they built it ; and if, as was frequently the case, the title of "palace" was chosen, a palace it remained, even although the facts which formed the building material were ill-burnt brick or mud, such as nothing better than a pigstye could be formed out of.

Very careful, very industrious, and very skilful workers think that minute bodies, of various shapes and sizes, which they call "germs," are the cause of some of the most serious and fatal diseases which afflict mankind. Consumption, it is believed, is caused by germs : and so too are typhoid fever, cholera, scarlet fever, diphtheria, and almost every disease which runs an orderly feverish course. If that be really so, then the destruction of the germs in their inactive condition should prevent the disease : their destruction in their active condition should cure it. In that case the doctor would have simplified his work very much. He would be master of the first condition of success—knowledge. He could affirm what the disease really is, and therefore exactly what he must do in order to prevent or cure it. But are germs really the cause of the diseases we have named, and of many others? That is a question the importance of which it is impossible to over-estimate, not only from the doctor's point of view who wishes to cure disease, but still more from the patient's, who is the subject of it, and every other mortal man's who may become the subject of it.

There is a strong consensus of opinion in Germany, France, and England that real ground has been gained ; that certainty has been arrived at ; that germs are in very truth the invisible but all-potent creatures which have hurried to an untimely grave "young men and maidens, old men and children," without discrimination and without remorse. But Dr. Semmola, a famous Italian who took part in the recent International Medical Congress at Washington, exploded some sceptical dynamite in its midst with disastrous results. He believes in the germs to a certain extent, but thinks it probable their potency has been much exaggerated, if not indeed often misunderstood. Says Dr. Semmola : "The chief error of to-day is that of bacteriology being the key to all pathology." Not merely a small mistake, but "the chief error of to-day." "We drink millions of microbes (bacteria) in our water, we respire millions in the air we inhale. Sometimes these microbes affect us, sometimes killing in a few hours . . . ." "It has at once been concluded that microbes are the cause of disease, whereas in many cases microbes are but the effects of disease. We ought to reproduce the disease artificially by means of microbes before definitely concluding that they are the cause. The experiments made do not give satisfactory results except in the instances of carbuncle and tuberculosis." That is one of the latest utterances of competent scientific authority. There are then two schools : those who believe in the universal potency of bacteria, and those who are waiting for more evidence. The latter are undoubtedly right, and display the scientific temper in its most perfect form.

In the meantime, what about the patient and the treatment he is to receive pending the solution of this, perhaps, interminable problem ? What a blessing it is that practice so often precedes theory in the order of development : that corn is grown and bread made before a science of agriculture can be formulated. The doctor's speculative brain may make him argue backwards and forwards about germs, as before he has argued about humours and solidism, but his genuinely human heart, which he carries with him to the bedside as well as his speculative head, makes him set himself with a will to do what he can to relieve or cure his patient by such means as knowledge and experience have taught him to rely upon. The settlement of the theoretical question can wait. Until he can settle it he will use the arts he has so often found successful before ; and though he may sometimes use them with an incomplete knowledge both of the disease and the mode of action of his remedy, yet will his assiduous and watchful care often pluck his patient from the very jaws of death. The baker makes bread which is the staff of life, and yet he seldom knows anything whatever about the chemical constitution of the flour, or the vegetable nature of the "Torula" which he calls "yeast." The sailor trims his sail and makes use of the slightest breeze from any quarter, but he knows no more about the composition of forces than he does about the coffin of Rameses or the height of the mountains of the moon.

We work with such knowledge as we can gain and with such tools as we have got, and it is wonderful what successful work can be turned out when the worker enters with his whole heart and soul into the difficulty. Patients need not fear to put themselves into the doctor's hands if they feel that they can rely upon his honesty and goodness as a man. His knowledge is more than sufficient to save him from bungling, and his sense of duty all-potent to prevent him from trying dangerous experiments upon them. Just as a guide who cannot read the directions on the milestones may take his party safely up the steepest and most dangerous mountains, so may a medical man who has neither the opportunity nor the capacity for deciding speculative questions, conduct a patient with the most finished skill and judgment through diseases which but for his care would inevitably end in death. Find a man in any calling whose honesty is undoubted and his abilities fair, and that is the man to trust in the hour of need.

